# The way forward to a renewed and improved *Health and Quality of Life Outcomes*

**DOI:** 10.1186/s12955-021-01746-4

**Published:** 2021-04-01

**Authors:** Mark Oremus, Oliver Rivero-Arias

**Affiliations:** 1 School of Public Health and Health Systems, University of Waterloo, Waterloo, ON Canada; 2 National Perinatal Epidemiology Unit (NPEU), Nuffield Department of Population Health (NDPH), University of Oxford, Oxford, UK

Over the last few months, *Health and Quality of Life Outcomes* has undergone a change in leadership with Mark Oremus (School of Public Health and Health Systems, University of Waterloo) and Oliver Rivero-Arias (Nuffield Department of Population Health, University of Oxford) being named Editors-in-Chief.

Our overall mission as Editors-in-Chief is to raise the scientific profile of the journal and improve the quality of accepted submissions through the implementation of a streamlined editorial process for authors, associate editors, and peer reviewers. This will allow published articles to have a higher impact on policy, practice, and patient outcomes.

We are taking the following steps to achieve our mission: (1) revising the journal’s aims and scope to clarify the types of manuscripts that will be considered for publication moving forward; (2) expanding the editorial board with expertise to align with the revised aims and scope of the journal; (3) introducing a triage process after initial submission, at the editor-in-chief level, to identify suitable manuscripts, thereby giving associate editors more quality time to spend on the peer review process; and (4) clarifying the instructions to authors and mandating the use of article reporting guidelines to promote transparency in research. We are hoping these steps will also improve the time between manuscript submission and final publication. Over the next few months, readers will see these changes coming into effect.

The biggest change will be a sharper focus on the publication of manuscripts that directly pertain to health-related quality of life, or HRQOL. We believe the value of the journal, and the scientific impact of the manuscripts published therein, will improve considerably if *Health and Quality of Life Outcomes* becomes the journal of choice for a narrower range of articles focused on HRQOL.

**Table:** 

Please note the revised aims and scope: *Health and Quality of Life Outcomes* will consider original research employing appropriate quantitative or qualitative methods; also included are systematic reviews/meta-analyses, protocols, commentaries on articles, and letters to the editor. We will welcome contributions in the following areas:
(1) hypothesis-based or theory-driven primary or secondary data analyses employing observational or experimental data, provided HRQOL information is the main focus of the manuscript and used as an independent or dependent variable
(2) systematic reviews or meta-analyses of HRQOL studies
(3) articles describing novel theoretical frameworks created to underpin the development or use of HRQOL instruments
(4) submissions describing the psychometric properties of instruments to measure HRQOL or similar patient-reported outcomes
(5) preference elicitation to estimate HRQOL value sets (e.g., health utilities)
(6) study protocols, provided data collection and analysis have not yet begun

To encourage the submission of high-quality work, all manuscripts submitted to the journal should conform to the relevant reporting guidelines (e.g., PRISMA, PRISMA-P, MOOSE, STROBE, CONSORT, etc.). Systematic reviews/meta-analyses and randomized controlled trials will only be considered for publication if they are preceded by a registry listing (e.g., PROSPERO, clinicaltrials.gov) or a published study protocol.

As the editorial board implements the new aims and scope, readers may notice the publication of articles that fall outside of the new requirements. These ‘legacy’ manuscripts are being published because the editors-in-chief did not want to adversely affect submissions that were already in peer review at the time of the change in focus.

The editorial board recognizes the international nature of research and encourages the submission of manuscripts from around the globe. However, to improve the peer review process and promote the timely disposition of manuscripts, *Health and Quality of Life Outcomes* will discourage authors from submitting manuscripts describing the validation of existing instruments in populations from highly-specific settings/contexts.

We believe the changes described above will produce a vigorous journal that is destined to have a strong impact on the scientific discourse about HRQOL for years to come.

Mark Oremus, PhD

Oliver Rivero-Arias, DPhil

Editors-in-Chief, Health and Quality of Life Outcomes

